# Sunproofed study protocol: A mixed-methods scoping study of sun safety policies in primary schools in Wales

**DOI:** 10.1371/journal.pone.0268141

**Published:** 2022-05-11

**Authors:** Julie Peconi, Claire O’Neill, Greg Fegan, Kirsty Lanyon, Timothy Driscoll, Ashley Akbari, Alan Watkins, Rachel Abbott

**Affiliations:** 1 Swansea Trials Unit, Medical School, Swansea University, Swansea, Wales, United Kingdom; 2 Population Data Science, Swansea University, Swansea, United Kingdom; 3 Cardiff and Vale University Health Board Cardiff, Cardiff, Wales, United Kingdom; UNITED KINGDOM

## Abstract

**Background:**

Skin cancer, including melanoma and non-melanoma (keratinocyte), is increasing in incidence in the UK. Accounting for half of all cancers in England and Wales, the disease significantly impacts overstretched dermatology services. Research suggests that 86% of melanoma is preventable with modified sun exposure. Educating children about sun safety in schools can help prevent skin cancer and is recommended by major health organisations. In England, teaching sun safety in primary schools is compulsory, while in Wales this is left to school discretion.

**Aims:**

Understand how primary schools in Wales are responding to growing skin cancer rates and explore the effectiveness of sun safety policies in schools on knowledge and behaviour.

**Methods:**

Sunproofed is a mixed-methods scoping study comprising 5 work packages (WP) using survey and routine electronic health record (EHR) data supplemented by qualitative case studies. Objective(s) are to: WP1: Discover if primary schools in Wales have sun safety policies; policy characteristics; determine factors that may influence their presence and identify areas where schools need support. WP2: Determine what EHR data is available regarding the incidence of sunburn in primary school children and the feasibility of using this data to evaluate the impact of sun safety policies. WP3: Understand the impact of sun safety policies on sun-safe knowledge and behaviour amongst children, parents, teachers, and school management; identify barriers and facilitators to schools implementing sun safety policies. WP4: Co-produce guidance regarding sun safety policies and best methods for implementation in schools. WP5: Disseminate guidance and findings widely to ensure impact and uptake.

**Discussion:**

Skin cancer rates are increasing in the UK, straining limited resources. Sunproofed has the potential to inform the development of future prevention activities, both in Wales and beyond. This could reduce the number of skin cancer cases in the future and keep people healthier for longer.

## 1. Introduction

### 1.A Background

Skin cancer, including melanoma and keratinocyte (non-melanoma), is now a worldwide health concern, and the United Kingdom (UK) is no exception. Since the early 1990s, melanoma skin cancer incidence rates have more than doubled (140%), and keratinocyte skin cancer rates have increased by 169% [1]. In practice, this translates to 46 new cases of malignant melanomas and 430 keratinocyte skin cancer cases in the UK every day (2016–2018) [1].

It is estimated that skin cancer now constitutes 50% of a dermatologist’s workload [2] and with a shortage of dermatologists in rural and remote areas, dermatology services in the UK continue to feel the pressures of this increasing workload [3]. The financial burden of skin cancer is pressing, with hospital episode statistics suggesting that the cost of skin cancer treatment was forecast to increase from between £106–£112 million in 2008 to at least £180 million in 2020 in England alone [4].

However, estimates suggest 86% of melanoma is preventable with modified sun exposure [5, 6]. Most skin cancers are the result of excess ultra-violet (UV) exposure, the majority occurring before adulthood [7] and severe sunburn as a child greatly increases the risk of developing future skin cancer [8]. Clearly, avoiding excess sun exposure is key when it comes to preventing skin cancer. While this sounds straightforward, in the UK, a face-to-face survey highlighted the fact that both knowledge and behaviour about how to reduce the risk of skin cancer risk are limited and needed to improve if skin cancer rates were to decrease. Respondents underestimated the importance of sunburn in the aetiology of skin cancer with only 40% rating it a very important risk factor. Similarly, while only 43% recognised the role of a high factor sunscreen in reducing their risks, even less, (one third) used sun cream to prevent their risks [9]. Additionally, individuals generally perceive their overall susceptibility to skin cancer as low [10] and both positive attitudes to tanning and perceived barriers to adopting sun-safe behaviours (e.g. the hassle of putting on sun cream) are both major obstacles in the battle for prevention [10].

Skin cancer prevention activities have been shown to be beneficial and effective when delivered to children both due to the importance of limiting sunlight exposure during childhood and because this is when individuals are more amenable to adopting new attitudes and behaviours [11]. Children spend almost half their childhood at school with approximately 8 school hours outside per week, often during the hours of the day (11am-3pm) and months of the year (May-July) where UV levels are at their highest in the UK. School-based education programmes are one way to instil lifelong sun-safe habits from an early age, with several education programmes being shown to be effective in children under 18 [12], and in primary schools [13]. While these interventions have been more successful in Australia than in North America and Europe [14], the evidence suggests more research is needed [12, 14]. In a critical review of ‘Skin Cancer Prevention for Children’, most programs improved sun safety knowledge, but changes in attitude and behaviours were smaller and strategies to improve sun safety policies needs further study [14].

In England, in 2019, sun safety education became a compulsory component of the curriculum. Similarly, the World Health Organisation [15]; the Melanoma Taskforce [16] and NICE [17] all recommend sun safety is taught in schools. However, in Wales it is still up to individual schools to implement their own policies and sun safety education is not mandatory. This is despite The National Assembly’s Children and Young People Committee’s 2012 inquiry into sun protection recommending schools had clear plans for sun protection and The Welsh Network of Healthy Schools National Quality award recommending schools have a sun safety policy in place.

While there is a clear case for focusing on skin cancer prevention in schools: a strong policy emphasis on prevention; health expert recommendations; the fact that 86% of melanomas are preventable and encouraging results from other studies on educational approaches to sun safety; there is no current funding from Welsh Government for skin cancer prevention, and little UK research in this area. To address this important gap, we have designed the Sunproofed study, a 2-year mixed-methods scoping study funded by Health and Care Research Wales (HCRW).

### 1.B Study aim

Understand how primary schools in Wales are responding to growing skin cancer rates and explore the effectiveness of sun safety policies in schools on knowledge and behaviour.

### 1.C Objectives

The study is divided into 5 work packages (WPs), each with its own objective(s). To:

1. Discover if primary schools in Wales have sun safety policies; policy characteristics; determine factors that may influence their presence and identify areas where schools need support. (WP1)
2. Determine what EHR data is available regarding the incidence of sunburn in primary school aged children and the feasibility of using this data to evaluate the impact of sun safety policies. (WP2)
3. Understand the impact of sun safety policies in schools on sun-safe knowledge and behaviour levels amongst children, parents, teachers, and school management; identify barriers and facilitators to schools implementing sun safety policies. (WP3)
4. Co-produce guidance regarding sun safety policies and best methods for implementation in schools. (WP4)
5. Disseminate guidance and findings widely to ensure impact and uptake. (WP5)

## 2. Design

### 2.A Overview

We will use the Behaviour Change Wheel as a theoretical framework [18]. This framework focuses on 3 essential conditions for behaviour change: capability, opportunity and motivation. It lays out how interventions such as education, training and modelling can change behaviour. We will also use the framework of co-production [19] to produce guidance on the best methods for schools to implement sun safety programmes.

**Fig 1:**
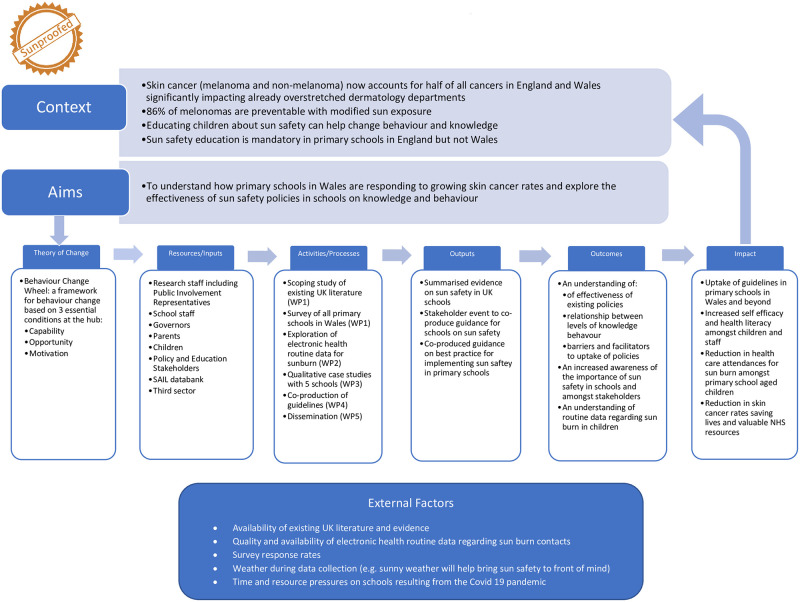
Sunproofed logic model.

To clarify how these theoretical assumptions, project inputs and processes link with our outcomes, we have created a logic model, [20] (Fig 1) a graphical representation of how we intend Sunproofed to work, an approach suggested by the MRC and supported by all major health promotion agencies [21, 22]. The logic model will be instrumental in ensuring we capture all inputs, outputs and any opportunities for transferability beyond the school setting.

### 2.B Public involvement

As schools operate under unique pressures, input from those who understand the complexities of the school environment was paramount in shaping this research. Two teacher representatives provided input from a school’s perspective into the study proposal. Going forward, they will help with the design of data collection tools and participant facing information, ensuring materials are written age appropriately. They will also be instrumental in the co-production of guidance regarding the implementation of sun safety policies, the final report and dissemination.

Feedback from charities, such as Tenovus Cancer Care, who previously worked with schools on sun safety, is that difficulties still exist in engaging schools despite making materials easily available. Our 2 teacher representatives will be instrumental in helping overcome this issue.

We will also recruit 2 parent representatives who will advise us on what’s important to them, including some of the challenges they face when sending their children to school on sunny days. In line with best practice, we will provide honoraria, briefings and other support as needed and report public involvement in our outputs [23–25].

## 3. Methods

As each of the 5 WPs is distinct in its objectives and methods, we present methods and consent procedures under each, separately defining the inclusion/exclusion criteria, setting, search strategy, sampling, data collection and analysis where relevant.

We have received ethical approval from the Swansea University Medical School Research Ethics Sub-Committee (ref- 2021–0096), and key team members have undertaken Disclosure and Barring Service (DBS) checks as necessary to allow them to work with children.

### 3.1 WP 1: Scoping study and nationwide school survey

#### Scoping study

We wish to better understand the current evidence for sun safety in UK schools. We will write a separate scoping study review protocol outlining our search, data extraction and reporting strategies. This protocol will include our comprehensive search strategy outlining key search terms as well as popular academic databases to be searched. We will search reference lists from included papers and contact colleagues, charities, and experts in this area for any relevant grey literature and unpublished reports. We will conduct this search at the beginning of the project and update it to include any new studies before compiling the evidence to inform the co-production of guidance. We will include interventions in primary schools only (secondary schools and nurseries are likely to have different outcomes), UK based studies only, English language studies only and those from 2000 onwards.

#### Nationwide school survey

We will survey all primary schools (n = ~1,225) in Wales to discover if schools currently operate sun safety policies and whether adoption of a sun safety policy varies by school characteristic including: geographical region, size, ethnicity, language and the proportion of children on free school meals. We will also use the survey to determine, what support, if any schools need in this area.

The questionnaire will be based on school surveys used in New Zealand [26], and in South Wales [27]. It will consist of tick-box questions such as ‘does the school have an existing sun safety policy’, ‘do teachers allow time for applying sunscreen’ and ‘does the school have outdoor shaded areas’. There will be a free text box to allow schools to express their ideas or concerns, but the questionnaire will be kept deliberately short to maximise response rates.

We will include an ethically approved email invitation letter and participant information sheet with the questionnaire and make it explicit that by completing the survey that the respondent is consenting to participate. We will also make it clear that they have the right to close the survey at any time and their results will not be saved.

To ensure credibility, we will engage with all 22 local authorities (LAs) in Wales to get their endorsement and look to use the LA, HCRW and Swansea University logos on our covering email. Schools will be given the opportunity to complete the survey in Welsh. All respondents will be entered into a draw to win £500 for their school. Short questionnaires, university badges and monetary incentives have all been shown to increase response rates [28]. Three reminder emails will be sent to non-responders. We will also include a tick box to indicate if the school would be interested in participating in further research allowing us to identify potential participants for WP3.

We will analyse survey results following a predefined analysis plan using simple statistics, such as measures of distribution means, cross-tabulations and tests of association such as t-tests for continuous variables and Pearson’s chi-square for categorical variables. Results will be presented by different stratifying criteria such as: geographical region, school size, language, ethnicity and the percentage of children receiving free school meals. We will use these criteria to identify schools for the qualitive case studies in WP3 to ensure we have a cross-section of schools represented. We will analyse free-text responses using thematic analysis following the recommended 6 step process: familiarisation, coding, generating themes, reviewing themes, defining and naming themes and writing up [29].

### 3.2 WP 2: Exploration of EHR data

In severe cases of sunburn in children, parents may ring National Health Service (NHS) Direct Wales, or the non-emergency number ’111’, present to their General Practice (GP), a minor injury unit (MIU), the Emergency Department or a Burns Unit. NHS contacts are routinely recorded and coded within electronic health records (EHRs). The Secure Anonymised Information Linkage (SAIL) Databank holds anonymised individual-level population-scale EHR data sources linkable across multiple data sources, enabling longitudinal research [30]. This project has been approved by the SAIL independent Information Governance Review Panel (IGRP), project 1080.

To date, the information in SAIL has not been used to explore the numbers of severe sunburn related incidents in children in Wales. We will aim to identify any links with healthcare contacts for sunburn and area-based information on schools, including their catchment areas (for example, will the data provide further evidence on the effectiveness of any schools’ sun safety policies?). We will explore how far back this data is available, assess data quality retrospectively and, recognising that a lack of admissions may be linked to poor weather, will look at seasonality over the years.

### 3.3 WP 3: Qualitative case studies

We will identify 5 schools from those indicating that they would like to participate in further research in WP1. The selection of schools will be informed by our logic model and sampling frame. The schools will be selected to ensure that at least some are operating a sun safety policy, one of which will be a Welsh Medium school if possible. The other schools will be chosen from those who do not have active policies or teaching in place. We will ensure that schools represent different percentages of children on free school meals, ethnicity, size, and location (e.g. coastal or inland) within this sample. Permission to conduct the case studies in a school will be given by the Headteacher or a delegate and confirmed in writing or electronically.

Within our 5 schools, we will conduct in-depth case studies with focus groups, telephone interviews and short questionnaire data. To allow schools to communicate in their preferred medium, a Welsh speaking Qualitative Researcher will conduct the case study in the Welsh Medium School. We will work closely with this researcher to ensure data collection activities are as similar as possible to the other 5 schools.

In each school, we will conduct:

- Focus groups with teachers (n = ~8–10 people per group) to gather their sun safety knowledge and behaviour and their opinions on their school’s current practices and policies. We will explore how effective the teachers see the policies in terms of helping them assist children, modify the school environment and what barriers or facilitators to implementation currently exist. Focus groups will be conducted at a convenient time and ideally will be face-to-face. However, as online methods are increasingly acceptable [31] and allow more people to speak [32] we will explore these if necessary.
- Semi-structured interviews with school governors who have specific responsibility for wellbeing if possible (1/school, n = 5). These will explore the feasibility of implementing sun safety policies in schools, including any barriers to action.
- Semi-structured telephone interviews with parents or guardians (~ 20 in total), drawn from all case study schools. The interviews will explore parents’ own knowledge and behaviours as well as their thoughts on sun safety policies in schools. While predicting numbers for qualitative data collection is difficult, we estimate 20 interviews to be sufficient to reach data saturation (e.g. no new themes emerging). We will monitor this by reviewing the codebook, particularly after 5, 10 and 15 interviews, to identify the point at which no new codes are developed.
- Short online questionnaire surveys with children in KS2 classes (years 3–6, n = ~20 classrooms). These surveys will be presented as ‘quizzes’ for the children and will consist of ~10 questions designed to measure sun safety knowledge and behaviour. PPI reps will help develop and pilot these questionnaires to be suitable for the children’s ages and literacy level. Parental consent will be sought in the first instance before gaining permission from the children themselves.
- A focus group with policymakers, healthcare professionals and education experts (n = ~7 in the group). The discussion will focus on how respondents feel about sun safety being taught in schools, the priority they attach to it and any potential barriers to implementing policies in schools.

#### Case study consent procedures

For the individual data collection activities within the school and for all other data collection activities, we will obtain formal informed consent from participants on research activity specific consent forms. Potential participants will be given full and accurate information about the research and be given at least 7 days to consider agreeing to be part of the study. Questions will be encouraged, and potential participants informed of their right to withdraw from the study at any point without the need for explanation.

For the students, we will first seek consent from their parents who will be given the option not to have their child participate. As parents and schools are very busy, we will use an opt-out process giving parents a specific date (at least 7 days in the future) to opt-out by. Once this has passed, we will seek assent from the student. Students’ assent will be taken before they complete the questionnaire and as part of the questionnaire itself.

All consent will be taken by the CI or a trained delegate. In as much as possible we will use e-consent procedures following Swansea Trial’s Unit Standard Operating Procedure (SOP). All electronic personal data will be stored in password protected documents on a secure Swansea University server. All hard copies will be stored as outlined in the SOP, in a separate physical location to any data collected.

#### Case study analysis

We will audio record all qualitative data with participant consent, and have data professionally transcribed. We will compare the transcripts against raw data for accuracy, ensuring identifiable data is removed. We will use NVivo12, a computer-assisted qualitative data analysis software package to undertake data analysis. This package also provides robust data management tools. We will analyse data using thematic analysis [29] and analyse survey data in SPSS using appropriate simple statistics. We will compare children’s results by class year and by school and whether that school currently operates a sun safety policy. We will explore differences between knowledge and reported behaviour to see whether there are any differences between aspiration, policy and practice. All data analysis will be undertaken in accordance with the principles of reliability and validity checking using the coder comparison query tool in NVivo12. A second qualitative researcher is included in the team to ensure this work is undertaken to a robust standard.

### 3.4 WP 4: Co-production of guidance

Hickey describes co-production as a process in which researchers, practitioners, and the public share power [19]. He argues that public participation in research generates useful knowledge leading to better outcomes. We will organise a stakeholder workshop to draw together findings from WP1-3 and co-produce guidance for schools on implementing sun safety policies and all study members and participants (teachers, heads of schools, governors, parents) will be invited to attend. Other key stakeholders, including charities, members of The National Assembly for Wales Children and Young People and the Education Committee will also be invited to attend. We recognise that it may be difficult for some participants (e.g. teachers) to attend and will explore the most effective way to elicit their opinions and make sure their ideas are included.

As bringing children to this event will be difficult, PPI representatives advised that we seek to involve children early on to participate in this co-production. We will explore the option of a smaller stakeholder event for children, perhaps with schools identifying their own child sun safety champions as ambassadors and co-producing documentation of their own that could feed into the larger stakeholder workshop. Children are integral to this process, and we will ensure that their views and voices are included.

### 3.5 WP 5: Dissemination and impact

We will produce both a study Publication and a Communications Plan. The Publication Plan will indicate key papers to publish, potential co-authors, target journals for submission and proposed dates for submission. It will also outline specific conferences and meetings we will look to present at, such as Public Health Wales’ annual conference, the British Association of Dermatologist’s Annual Meeting and the Cross-Party Group on Skin. The Communication Plan will clearly outline how we will give feedback to the schools and other members of the public, including dissemination of the co-production of guidance. Within this plan, we will also detail methods for engaging with the media and communicating with other key stakeholders such as policymakers and skin cancer experts.

## 4. Discussion

Rates of skin cancer are increasing in Wales, increasing the strain on already limited NHS resources. With strong evidence that reducing sun exposure in children can help prevent skin cancer in later life, an urgent shift from treatment to prevention is needed, and education in schools is one way to facilitate this. Therefore, it is essential that we understand the current situation in Wales and what can be done to improve it. The evidence produced by this first national scoping study of sun safety policies in primary schools in Wales has the potential to benefit children and their parents and to inform the development of future practice moving toward prevention over intervention.

To ensure the study is successful, we will manage our timelines to coincide with important dates in the school calendar and will undertake data collection activities as much as possible when sun safety is more likely to be front of mind. We will also remain mindful that schools are under great pressure and are often operating with limited time and resources. Therefore, we will ensure we do not place any additional burdens on schools by ensuring research methods are manageable, meaningful and flexible. Additionally, the curriculum for Wales 2022 requires teachers to take an active role in research. To ensure the research is relevant for staff, this study links to a key aim of the new curriculum: to develop children as healthy individuals who make informed decisions about their health. With sun safety now part of the curriculum in England, we will monitor the situation closely and consider any early findings here as our study develops. This evidence will also help resource and time-strapped schools with local decision making by providing them with best practice advice, thus improving service delivery and outcomes.

By sharing this study protocol, we can support Wales’ move towards prevention and helping individuals avoid future skin cancer, thus improving their health and wellbeing and saving valuable NHS resources.
